# Plasma metabolites of aromatic amino acids associate with clinical severity and gut microbiota of Parkinson’s disease

**DOI:** 10.1038/s41531-023-00612-y

**Published:** 2023-12-14

**Authors:** Szu-Ju Chen, Yu-Jun Wu, Chieh-Chang Chen, Yu-Wei Wu, Jyh-Ming Liou, Ming-Shiang Wu, Ching-Hua Kuo, Chin-Hsien Lin

**Affiliations:** 1 Department of Neurology, National Taiwan University Hospital, College of Medicine, National Taiwan University, Taipei, Taiwan; 2 Department of Neurology, National Taiwan University Hospital Bei-Hu Branch, Taipei, Taiwan; 3 Graduate Institute of Clinical Medicine, College of Medicine, National Taiwan University, Taipei, Taiwan; 4 School of Pharmacy, College of Medicine, National Taiwan University, Taipei, Taiwan; 5 Division of Gastroenterology and Hepatology, Department of Internal Medicine, National Taiwan University Hospital, College of Medicine, National Taiwan University, Taipei, Taiwan; 6 Graduate Institute of Biomedical Informatics, College of Medical Science and Technology, Taipei Medical University, Taipei, Taiwan; 7 The Metabolomics Core Laboratory, NTU Centers of Genomic and Precision Medicine, National Taiwan University, Taipei, Taiwan; 8 Department of Pharmacy, National Taiwan University Hospital, Taipei, Taiwan; 9 Institute of Molecular Medicine, College of Medicine, National Taiwan University, Taipei, Taiwan

**Keywords:** Parkinson's disease, Biomarkers

## Abstract

Gut microbial proteolytic metabolism has been reportedly altered in Parkinson’s disease (PD). However, the circulating aromatic amino acids (AAA) described in PD are inconsistent. Here we aimed to investigate plasma AAA profiles in a large cohort of PD patients, and examine their correlations with clinical severity and gut microbiota changes. We enrolled 500 participants including 250 PD patients and 250 neurologically normal controls. Plasma metabolites were measured using liquid chromatography mass spectrometry. Faecal samples were newly collected from 154 PD patients for microbiota shotgun metagenomic sequencing combined with data derived from 96 PD patients reported before. Data were collected regarding diet, medications, and motor and non-motor symptoms of PD. Compared to controls, PD patients had higher plasma AAA levels, including phenylacetylglutamine (PAGln), p-cresol sulfate (Pcs), p-cresol glucuronide (Pcg), and indoxyl sulfate (IS). Multivariable linear regression analyses, with adjustment for age, sex, and medications, revealed that the plasma levels of PAGln (coefficient 4.49, 95% CI 0.40–8.58, *P* = 0.032) and Pcg (coefficient 1.79, 95% CI 0.07–3.52, *P* = 0.042) positively correlated with motor symptom severity but not cognitive function. After correcting for abovementioned potential confounders, these AAA metabolites were also associated with the occurrence of constipation in PD patients (all *P* < 0.05). Furthermore, plasma levels of AAA metabolites were correlated with the abundance of specific gut microbiota species, including *Bacteroides sp. CF01-10NS*, *Bacteroides vulgatus*, and *Clostridium sp. AF50-3*. In conclusion, elevated plasma AAA metabolite levels correlated with disease characteristics in PD, suggesting that upregulated proteolytic metabolism may contribute to the pathophysiology of PD.

## Introduction

Parkinson’s disease (PD) is a common neurodegenerative disease characterized by various progressive motor and non-motor symptoms^1^. Neuropathological evidence reveals that, at least in some patients, the pathognomonic PD pathology termed the Lewy body may initiate from the enteric nervous system, and then propagate to the brain via cell-to-cell transmission^2^. The onset of gastrointestinal dysfunction, especially constipation, may precede the motor symptoms of PD by decades^3^. Many reports have shown altered gut microbiomes in PD patients compared to unaffected controls^4^. These observations suggest that gut microenvironment alterations may trigger the PD process though the complicated gut–brain axis^5^. The gut microbiota produces thousands of small molecules and metabolites that accumulate in the gastrointestinal system or reach distant organs through systemic circulation. Intestinal microorganisms interact with the host through secreted metabolites that act as signaling molecules in host–microbiome crosstalk, potentially modulating immune responses, endocrine secretion, and neurotransmission^5^. Understanding of how gut metabolites affect gut microbiome–host interactions in the PD process could shed light for future developing gut microbiome-based therapeutic interventions for PD.

The gut microbiota impact host metabolism by contributing enzymes, which are not encoded by the human genome, for the breakdown of dietary components. For example, the gut microbiota can fermentation carbohydrates to short chain fatty acids, proteins to aromatic amino acid (AAA) metabolites, lipids to bile acids and can synthesize certain vitamins, notably vitamin K and B. AAA metabolites—including phenylalanine (PHE), tyrosine, and tryptophan, have been reported to regulate immune, inflammatory, metabolic, and neuronal responses in the gut and brain^6^. Through redox pathways, PHE can be catabolized into phenylacetic acid (PAA), phenylpropionic acid (PPA), and phenyllactic acid (PLA)^6^. Furthermore, absorbed PAA can be conjugated by glutamine in the liver to form phenylacetylglutamine (PAGln). The dopamine precursor tyrosine is degraded by anaerobic bacteria to form p-cresol, which is metabolized into p-cresol sulfate (Pcs) and p-cresol glucuronide (Pcg) in the liver. These molecules are mainly excreted in urine, and are considered uremic toxins that promote inflammation and generate oxidative stress^6^. In contrast, indole derivatives produced from tryptophan by colonic bacteria generally have beneficial anti-inflammatory and anti-oxidative effects—with the exception of indoxyl sulfate (IS), which is a uremic toxin^6^. An aberrant degradative pathway of AAA is associated with several systemic disorders, including chronic renal disease, cardiovascular disorder, and neurodegeneration, including PD^7,8^. Some studies show higher concentrations of AAA metabolites, including PAGln and p-cresol, in the plasma and cerebrospinal fluid (CSF) of PD patients^9,10^; however, contradictory results exist^11,12^. Of note, a recent study observed reduced plasma AAA profiles in PD patients compared to controls and, furthermore, the levels were negatively correlated with the motor symptom severity of PD^11^.

In addition, some AAA metabolites, such as phenylalanine and phenylacetic acid, also have direct dietary sources and not solely derived from gut microbiota fermentation. Phenylalanine is present in a variety of protein-rich foods, including milk, eggs, and meat. It is also available as a dietary supplement and the artificial sweetener aspartame also contains phenylalanine. Phenylacetic acid, characterized by its sweet and floral flavors, can be detected in various food items such as hyssop, cowpea, endive, and shea tree. Hence, in the current study, we comprehensively examined plasma AAA levels in a large cohort of PD patients and unaffected controls matched for age, sex and diet habit. We further investigated how these AAA levels were associated with clinical severity (both motor and non-motor aspects), and gut microbiota changes, to delineate amino acid metabolism in the PD process.

## Results

This study enrolled a total of 500 participants, including 250 PD patients (67.4 ± 7.7 years old, 65.6% male) and 250 normal controls (67.4 ± 7.5 years old, 66.0% male). The participants’ clinical characteristics are listed in Table 1. The groups did not significantly differ in demographic distribution or laboratory results, including age, sex, body mass index, dietary pattern, medical co-morbidities, serum creatinine levels and dietary intake patterns measured by using the FFQ (Supplementary Table 1). MMSE scores were higher in control participants compared to PD patients (*P* < 0.01). Constipation was significantly more frequent in PD patients than controls (52% vs 41%, *P* = 0.01). In the PD group, the average Hoehn-Yahr stage was 3.2 ± 0.9 and mean MDS-UPDRS part III motor score was 35.0 ± 9.9. All PD patients received dopaminergic medication, with an average LEDD of 649.2 ± 206.0 mg/day.

**Table 1:** Clinical characteristics and plasma concentrations of microbial metabolites in participants.

	Healthy controls (*N* = 250)	PD patients (*N* = 250)	*P-*value
Age (years)	67.4 ± 7.5	67.4 ± 7.7	0.95
Sex (male), n (%)	165 (66.0)	164 (65.6)	0.93
BMI	22.6 ± 1.4	22.6 ± 1.5	0.99
Constipation, n (%)	103 (41.2)	130 (52.0)	0.01^*^
Creatinine (mg/dL)	0.9 ± 0.3	0.9 ± 0.2	0.96
Disease duration (years)	N.A.	7.4 ± 2.4	
Hoehn-and-Yahr stage	N.A.	3.2 ± 0.9	
Early-stage, n (%)	N.A.	46 (18.4%)	
Advanced-stage, n (%)	N.A.	204 (81.6%)	
MDS-UPDRS part III	N.A.	35.0 ± 9.9	
MMSE	28.7 ± 1.8	26.5 ± 2.7	<0.01^**^
LEDD	N.A.	649.2 ± 206.0	
Plasma metabolites (ng/mL)			
Phenylalanine (PHE)	9733.7 ± 1868.1	9674.9 ± 2252.9	0.50
Phenylacetic acid (PAA)	81.9 ± 106.1	113.7 ± 82.9	<0.001^***^
Phenylacetylglutamine (PAGln)	849.1 ± 2778.0	1024.3 ± 1025.6	<0.001^***^
Phenyllactic acid (PLA)	26.7 ± 15.1	30.3 ± 18.7	0.02^*^
Phenylpropionic acid (PPA)	28.8 ± 40.6	61.5 ± 122.7	<0.001^***^
P-cresol sulfate (Pcs)	2110.7 ± 2603.1	3265.1 ± 2899.3	<0.001^***^
P-cresol glucuronide (Pcg)	25.2 ± 101.8	41.5 ± 61.5	<0.001^***^
Indoxyl sulfate (IS)	1059.8 ± 2838.7	1132.2 ± 1380.6	<0.001^***^
Medical co-morbidity, n (%)			
Diabetes mellitus	61 (24.4)	49 (19.6)	0.19
Hypertension	68 (27.2)	53 (21.2)	0.12
History of cardiovascular disease	40 (16.0)	31 (12.4)	0.25
History of stroke	32 (12.8)	23 (9.2)	0.19

Variables are expressed as mean ± standard deviation or number (percentage).

*BMI* body mass index, *IS* indoxyl sulfate, *LEDD* levodopa equivalent daily dose, *MDS-UPDRS* Movement Disorder Society Unified PD Rating Scale, *MMSE* Mini-Mental State Examination, *N.A.* not available, *Pcg* p-Cresol glucuronide, *Pcs* p-Cresol sulfate, *PAA* phenylacetic acid, *PAGln* phenylacetylglutamine, *PHE* phenylalanine, *PLA* phenyllactic acid, *PPA* phenylpropionic acid. *P < 0.05; **P < 0.01; ***P < 0.001.

### Plasma levels of AAA metabolites in PD patients and controls

**Fig. 1:**
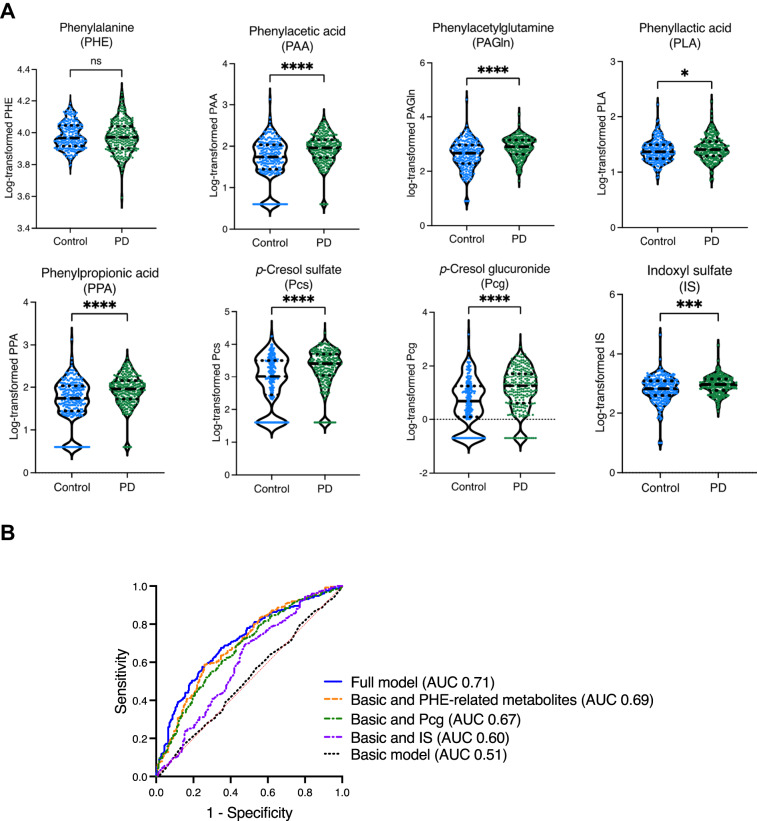
Comparison of microbial metabolites between patients with PD and healthy controls, and receiver operating characteristic (ROC) curve of microbial metabolites discriminating the PD and control groups. **A** Violin plot shows plasma concentrations of microbial metabolites, with the solid and dashed horizontal lines representing the median value and interquartile range, respectively. **B** The accuracy of PD prediction with various models including age, sex, and/or different microbial metabolites. AUC, area under the receiver operating characteristic curves. ns, non-specific, *P < 0.05, **P < 0.01. ***P < 0.001.

Compared to control participants, PD patients exhibited higher plasma concentrations of PHE oxidative metabolites, including PAA (113.7 ± 82.9 vs 81.9 ± 106.1 ng/mL, *P* < 0.001) and the downstream metabolite PAGln (1024.3 ± 1025.6 vs 849.1 ± 2778.0 ng/mL, *P* < 0.001); other PHE-derived products, including PLA (30.3 ± 18.7 vs 26.7 ± 15.1 ng/mL, *P* = 0.015) and PPA (61.5 ± 122.7 vs 28.8 ± 40.6 ng/mL, *P* < 0.001); tyrosine-related metabolites, including Pcs (3265.1 ± 2899.3 vs 2110.7 ± 2603.1 ng/mL, *P* < 0.001) and Pcg (41.5 ± 61.5 vs 25.2 ± 101.8 ng/mL, *P* < 0.001); and the tryptophan derivative IS (1132.2 ± 1380.6 vs 1059.8 ± 2838.7, *P* < 0.001) (Table 1, Fig. 1A). We found that age was not correlated with levels of the individual AAA subtypes in either the control or PD group (Supplementary Fig. 1A, B).

We next examined whether plasma concentrations of AAA metabolites could distinguish between PD patients and normal controls. Multivariable logistic regression models, adjusted for age and sex, revealed that PD occurrence was associated with higher plasma levels of PAA (OR 4.00, 95% CI 2.52–6.53, *P* < 0.001), PAGln (OR 3.73, 95% CI 2.43–5.84, *P* < 0.001), PLA (OR 3.03, 95% CI 1.25–7.50, *P* = 0.015), PPA (OR 1.75, 95% CI 1.36–2.27, *P* < 0.001), Pcs (OR 2.29, 95% CI 1.74–3.07, *P* < 0.001), Pcg (OR 2.07, 95% CI 1.65–2.61, *P* < 0.001), and IS (OR 3.02, 95% CI 1.79–5.24, *P* < 0.001) (Supplementary Table 2). ROC curve analysis was performed to evaluate the accuracy of predicting PD occurrence based on plasma AAA metabolites. The prediction accuracy, expressed as the AUC, improved from 0.51 in the basic model (age and sex; 95% CI 0.46 – 0.56, *P* = 0.745), and improved to 0.69 (95% CI 0.55–0.65, *P* < 0.001) with addition of the PHE-associated metabolites PHE, PAGln, PLA, and PPA (Fig. 1B). The AUC further improved to 0.71 (95% CI 0.66–0.75, *P* < 0.001) in the full model containing age, sex, and all AAA proteolytic metabolites, including PHE, PAGln, PLA, PPA, Pcg, and IS (Fig. 1B).

### Plasma levels of AAA metabolites and clinical severity of PD

**Fig. 2:**
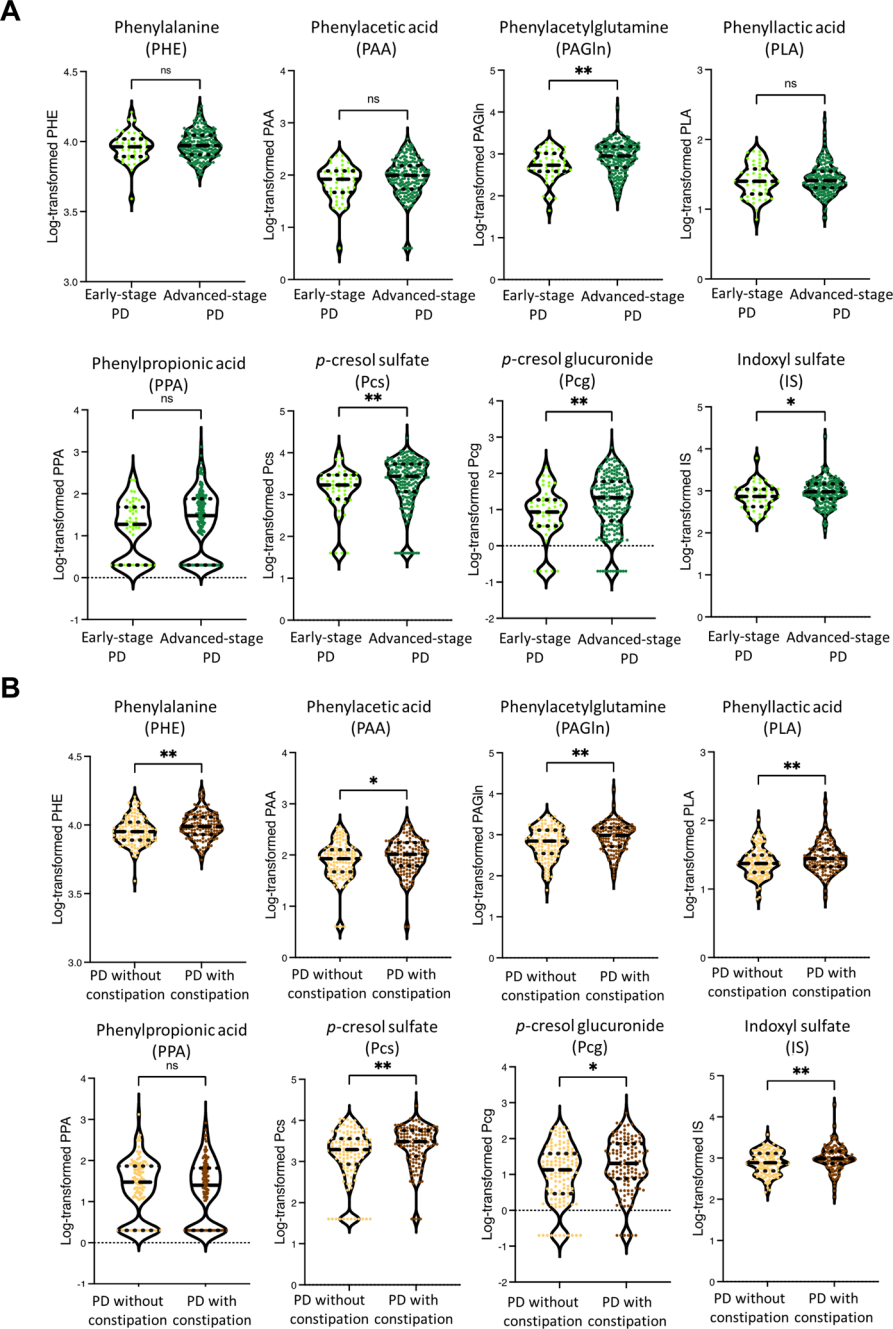
Comparison of microbial metabolites between PD patients with different clinical characteristics. **A** Violin plot shows the plasma levels of microbial metabolites in patients with early-stage and advanced-stage PD. Solid and dashed horizontal lines represent the median value and interquartile range, respectively. **B** Violin plots illustrate the comparison of plasma levels of microbial metabolites between PD patients with and without constipation. Solid and dashed horizontal lines indicate the median value and interquartile range, respectively. ns, non-specific. The comparison was conducted employing the Student’s *t*-test. *P < 0.05, **P < 0.01. ***P < 0.001.

Next, we examined whether AAA-related metabolites in systemic circulation reflected disease severity. Compared to patients with early-stage PD, those with advanced-stage PD exhibited higher plasma concentrations of PAGln (1100.0 ± 1100.2 vs 688.3 ± 467.7 ng/mL, *P* = 0.002), Pcs (3518.1 ± 3006.3 vs 2143.0 ± 2040.8 ng/mL, *P* = 0.003), Pcg (46.3 ± 65.8 vs 19.8 ± 29.1 ng/mL, *P* = 0.006), and IS (1177.5 ± 1466.4 vs 931.4 ± 892.1 ng/mL, *P* = 0.018) (Fig. 2).

Since antiparkinsonian medications were reported to affect the gut microbiota structure and microbial metabolite concentrations^13,14^, we examined the potential effects of antiparkinsonian medication on plasma levels of AAA metabolites. Spearman’s rank correlation test showed that most AAA metabolites had some degree of positive association with LEDD (Supplementary Fig. 2A). We further explored which types of antiparkinsonian medications affected plasma AAA levels. Among PD patients, users of dopaminergic agents and trihexyphenidyl exhibited lower plasma levels of PAGln, Pcs, Pcg, and IS compared to non-users (all *P* < 0.05), while users of catechol-O-methyltransferase inhibitor and amantadine had increased levels of PAGln, PAA, Pcs, Pcg, and IS compared to non-users (all *P* < 0.001) (Supplementary Table 3). Therefore, we adjusted for antiparkinsonian medication usage in the following further analyses.

Individual multivariable linear regression models analysing plasma levels of individual AAA metabolites and clinical severity adjusted for age, sex, occurrence of constipation, and anti-parkinsonian medication usage revealed that MDS-UPDRS part III motor score positively correlated with plasma concentrations of PAGln (coefficient 4.49, 95% CI 0.40–8.58, *P* = 0.032) and Pcg (coefficient 1.79, 95% CI 0.07–3.52, *P* = 0.042) (Supplementary Table 4 and summarized in Table 2). Consistently, further scatter plot analyses revealed that MDS-UPDRS part III motor scores significantly correlated with plasma levels of PAGln (*ρ* = 0.183, *P* = 0.004), PAA (*ρ* = 0.135, *P* = 0.033), Pcs (*ρ* = 0.165, *P* = 0.009), Pcg (*ρ* = 0.163, *P* = 0.010) and IS (*ρ* = 0.181, *P* = 0.004) (Supplementary Fig. 2B). However, spearman’s rank correlation analysis only showed that MMSE score tended to be inversely associated with plasma levels of PAGln (*ρ* = −0.124, *P* = 0.05) and IS (*ρ* = − 0.145, *P* = 0.02). After adjusting for age, sex, the occurrence of constipation, and anti-parkinsonian medication usage revealed that MMSE scores were not correlated with plasma concentrations of AAA metabolites (Table 2). The effects of other covariates, including age, sex, constipation, and anti-parkinsonism medications in the multiple linear regression analyses for individual AAA metabolite level and clinical disease severity were shown in the Supplementary Table 4.

**Table 2:** Summary of association results between individual plasma levels of AAA metabolites and clinical severity of PD in both motor and cognitive function.

		MDS-UPDRS part III scores				MMSE scores		
	Coefficient	95% CI	Cohen’s f^2^	*P*-value	Coefficient	95% CI	Cohen’s f^2^	*P*-value
PHE	9.33	−3.04, 21.70	0.11	0.139	−2.20	−5.51, 1.12	0.09	0.193
PAGln	4.49	0.40, 8.58	0.12	0.032^*^	−0.14	−1.19, 1.94	0.08	0.806
PLA	2.08	−3.77, 7.92	0.10	0.485	0.37	−0.87, 2.19	0.08	0.641
PAA	1.04	−2.91, 4.98	0.10	0.605	0.15	−0.90, 1.21	0.08	0.777
PPA	0.31	−1.41, 2.03	0.10	0.722	0.01	−0.45, 0.47	0.08	0.965
Pcs	2.06	−0.27, 4.39	0.11	0.083	0.15	−0.47, 0.78	0.08	0.630
Pcg	1.79	0.07, 3.52	0.12	0.042^*^	0.08	−0.39, 0.55	0.08	0.735
IS	3.98	−0.67, 8.64	0.11	0.093	−0.59	−1.85, 0.66	0.08	0.351

Multivariable linear regression models were applied to analyze the associations between microbial metabolites and disease severity. Motor severity measured by MDS UPDRS part III motor score or cognitive function examined by MMSE scale was set as dependent factor. The independent variables are age, sex, the occurrence of constipation, usage of antiparkinsonian medication including dopaminergic supplement, catechol-O-methyltransferase, monoamine oxidase-B inhibitor, amantadine and trihexyphenidyl, and log-transformed plasma metabolites.

*CI* confidence interval, *IS* indoxyl sulfate, *MDS-UPDRS* Movement Disorder Society Unified PD Rating Scale, *MMSE* Mini-Mental State Examination, *Pcg* *p*-Cresol glucuronide, *Pcs* *p*-Cresol sulfate, *PAA* phenylacetic acid, *PAGln* phenylacetylglutamine, PHE phenylalanine, PLA phenyllactic acid, PPA phenylpropionic acid.

*P < 0.05; **P < 0.01; ***P < 0.001.

### Plasma AAA metabolite levels and constipation symptoms

Constipation is the most common non-motor symptom in PD, and may affect gut microbiota composition and the gut metabolites in systemic circulation. In the control group, we found that the plasma levels of AAA metabolites did not significantly differ between constipated and non-constipated participants (Supplementary Fig. 3). However, PD patients with constipation had higher plasma levels of individual AAA metabolites than PD patients without constipation (all *P* < 0.01, Fig. 2B). After adjustment for age, sex, disease severity and LEDD, constipation remained significantly associated with higher plasma concentrations of PHE (*P* = 0.02), PAGln (*P* = 0.03), PLA (*P* = 0.02), Pcs (*P* = 0.008) and IS (*P* = 0.03) within the PD group (Supplementary Table 5).

### Plasma AAA levels correlate with gut microbiome species

**Fig. 3:**
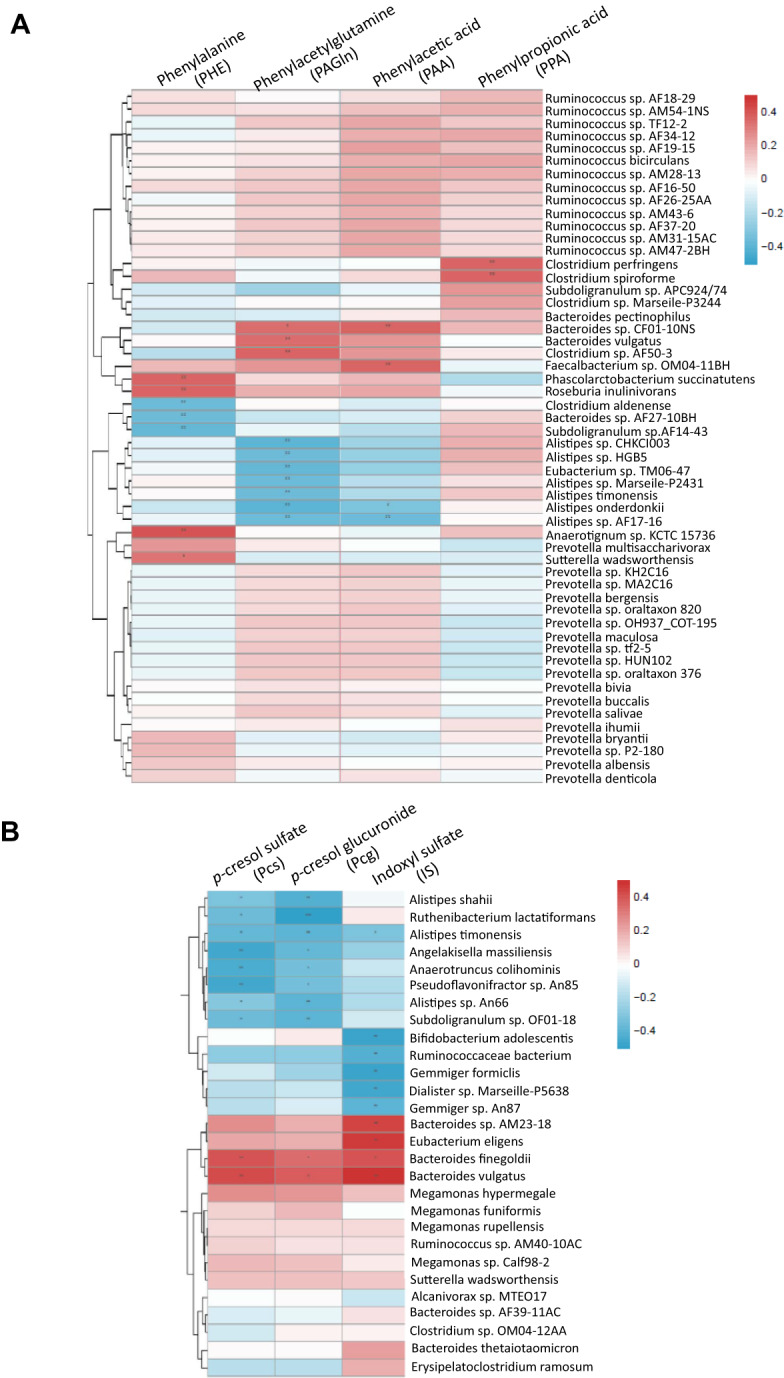
Heat-maps display the results of Spearman’s rank correlations test of the associations between microbial metabolites and the relative abundance of colonic bacteria. **A** Individual phenylalanine-related microbial metabolites, including phenylacetic acid (PAA), phenylpropionic acid (PPA) and phenylacetylglutamine (PAGln), are correlated with specific bacterial species. **B** Tryptophan and indole derivative-metabolites, including *p*-cresol sulfate (Pcs), *p*-cresol glucuronide (Pcg) and Indoxyl sulfate (IS), are correlated with specific bacterial species. The *r* values are represented by gradient colors, with red cells indicating positive correlations, and blue cells indicating negative correlations. Gut microbiota analysis was only performed in patients with PD. Among 250 PD patients, the fecal shotgun metagenomic data derived from 96 PD patients was reported before^35^. The correlations between the relative abundance of gut microbiota and plasma AAA levels were performed using Spearman’s rank sum tests with Benjamini and Hochberg adjustment for multiple tests. *P < 0.05, **P < 0.01. ***P < 0.001.

We next generated heat-maps depicting the Spearman correlations between the relative abundance of different bacterial species and individual AAA subtypes in plasma. We found that PAGln level was positively associated with the abundance of *Bacteroides sp. CF01-10NS*, *Bacteroides vulgatus*, and *Clostridium sp. AF50-3*, and negatively correlated with the abundance of *Alistipes* species (Fig. 3A). Plasma PAA, which is in the same pathway as PAGln, had similar associations with abundances of similar bacterial species, and was also positively correlated with abundance of *Faecalibacterium sp. OM04-11BH*. Another PHE reductive product, PPA, was positively related to abundance of *Clostridium perfringens* and *Clostridium spiroforme*. Plasma Pcs and Pcg are reportedly produced by similar bacterial species as PAGln^15^. Consistently, we found that plasma levels of Pcs and Pcg were positively correlated with the abundance of *Bacteroides vulgatus* and *Bacteroides finegoldii*, and negatively correlated with various *Alistipes* species. *Bacteroides* species were also positively correlated with plasma IS level (Fig. 3B). Together, these findings indicated that altered abundance of specific gut microbiota species were correlated with plasma levels of AAA metabolites, which were associated with PD clinical severity.

## Discussion

In a large cohort of participants, we performed simultaneous analyses of various AAA metabolites in systemic circulation, and examined their associations with gut microbiota detected by shotgun metagenomic sequencing, to comprehensively delineate the alterations of microbial AAA metabolites in relationship to PD occurrence and severity. We found that PD patients had higher plasma concentrations of AAA metabolites compared to controls. Moreover, among PD patients, these AAA metabolite levels were higher in patients with constipation than those without constipation. After adjustment for confounders, the increased plasma levels of PAGln and Pcg were correlated with motor symptom severity, and associated with specific gut microbiota changes.

PAGln level was increased in PD patients compared to controls, and was correlated with motor symptom severity after adjustment for confounders. The PAGln precursor PAA was also elevated in PD patients. PAGln is a conjugated product of glutamate and PAA produced from PHE through gut microbial proteolytic processes associated with the commensal bacteria *Clostridium*, which was detected in our cohort^16^. Elevated plasma PAGln levels are reported in systemic and neurological diseases, including chronic renal disease, cardiovascular syndrome, and diabetic neuropathy^16^. In line with our findings, two recent untargeted metabolomics studies with large multi-cohort study designs also revealed elevated plasma levels of PAGln in PD patients compared to controls and other neurological diseases^9,10^. However, some studies have not detected different plasma PAGln levels between PD patients and controls^11,12^. These inconsistent results may be explained by diet habits and medical co-morbidities, since PAGln is a deleterious biomarker for cardiovascular disorder, DM, and chronic renal failure^16^. Therefore, a control group well-matched for age, sex, and these other potential confounders is critical for elucidating the role of PAGln in the PD process. In our current study, the PD and control groups were matched for these confounders, and anti-parkinsonian medications were considered as an adjustment covariate in the analyses. Our results suggested that PAGln and its related pathway may be a mechanistic link in the PD process. Notably, recent functional studies show that PAGln—which is upregulated in PD patients and promotes PD neuropathology through neuroinflammation in PD rodent models—exacerbates inflammatory cascades by activating toll-like receptor 4-associated pathways^17^. Furthermore, PAGln administration in a mouse model exacerbates colitis by upregulating coagulation-related biological processes, and plasma PAGln level is elevated in patients with Crohn’s disease^18,19^. Crohn’s disease is associated with PD, since both diseases shared a common genetic risk factor, *leucine-rich repeat kinase 2* (*LRRK2*), as discovered in genome-wide association studies^20^. A recent epidemiology meta-analysis study demonstrated that Crohn’s disease patients have an 28% increased risk of PD compared to controls^21^. Furthermore, PAGln can cross the blood–brain barrier and exert neurotoxicity, thereby impairing neuronal activity^22^. These observations and our present results suggest that plasma PAGln level is a novel gut-oriented biosignature for PD. Further studies are warranted to investigate the molecular mechanism of PAGln in PD pathophysiology.

Our results also showed elevated plasma levels of Pcs and Pcg in PD patients compared to controls, which is in line with a recent untargeted metabolomic study^23^. Pcs and Pcg are both conjugated metabolites of p-cresol, a deleterious bacterial degradative product from tyrosine^6^. Elevated Pcs levels in CSF and plasma have been reported in several neurological disorders, including PD, Alzheimer’s disease, and multiple sclerosis^24^. Pcs impairs neuronal physiological function and exerts cytotoxic effect through generation of oxidative stress and upregulation of pro-inflammatory cytokines^22^. Pcg and Pcs have different biological functions. Some studies have reported that Pcg can prevent lipopolysaccharide-induced blood–brain barrier disruption^25^, while others show that Pcg suppresses mitochondrial function and aggravates the Pcs-induced oxidative burst of leukocytes, synergistically promoting inflammatory responses^26^. We observed that plasma Pcg level positively correlated with motor symptom severity in PD patients. Functional studies are needed to explore the molecular mechanisms underlying how Pcs and Pcg participate in the PD disease mechanism.

Constipation is among the most common non-motor and prodromal PD symptoms, and exhibits increasing prevalence with PD progression^27^. A major cause of constipation in PD is prolonged colonic transition time, likely resulting from α-synuclein accumulation in the enteric nervous system^27^. Impaired colonic peristalsis with delayed transit time is associated with gut dysbiosis, which results in increased colonic protein fermentation and a shift of the colonic microbiota metabolism away from carbohydrate fermentation and toward proteolysis^15^. In previous studies, more abundant *Ruminoccoccaceae*, *Oscillospira*, *Lachnospiraceae*, and *Clostridiales*, and higher plasma PAGln and Pcs levels, are observed in patients with impaired colonic motility, and are correlated with severity of constipation in PD^9,15^. Accordingly, we found that plasma PAGln and Pcs levels were higher in constipated than non-constipated PD patients, after adjustment for potential confounders, reinforcing that the colonic metabolism was skewed towards proteolysis in PD. In our cohort, these AAA proteolytic metabolites were associated with specific microbial species that have pro-inflammatory effects. Higher levels of PAGln, Pcs, Pcg, and IS were all associated with increased abundance of *Bacteroides* species, including *Bacteroides vulgatus*, which is a commensal bacteria that induces colitis in rodent models and is positively correlated with severity of inflammatory bowel disease^28,29^. More abundant *Bacteroides* species have also been reported in PD, although though are some conflicting results^30^. Low-grade intestinal inflammation could lead to intestinal epithelial barrier dysfunction and increased infiltration of luminal microbial metabolites, including AAA derivatives, into systemic circulation, which may partially explain the increased plasma AAA metabolites in our cohort^31^. Although we observed elevated plasma AAA levels in PD patients compared to controls, especially among those with advanced-stage PD and those with constipation symptoms, we did not find that plasma AAA concentrations were correlated with MMSE scores. One possible reasons is that the cognitive function of most of our enrolled PD patients did not reach dementia status, with a mean MMSE of >26. Another possible explanation may reflect the diverse pathophysiologic processes of motor and cognitive decline in PD^32^. Further studies enrolling PD patients with different severity of cognitive dysfunction are needed to explore the role of plasma AAA levels in PD dementia.

To our knowledge, our study is the largest cohort to comprehensively investigate the composition of AAA proteolytic metabolites in a cohort of PD patients and well-matched controls, and delineate their associations with gut microbiota. However, our study has several limitations. First, the cross-sectional study design limits our inference of causal relationship of AAA metabolite changes and PD. In addition, constipation symptoms in the current study may come from heterogeneous etiologies, including as a prodromal symptom of PD process, a co-morbidity due to motor dysfunction and gastrointestinal side effects of anti-parkinsonism medications (ex: anti-cholinergic agents and dopamine agonizts). A future prospective follow-up study including patients in the prodromal stage and drug naive patients are needed to correlate the changes of gut microbiota, plasma AAA levels and the symptoms of constipation. Further in vitro and in vivo assays are also needed to provide molecular insights into AAA metabolites in PD pathophysiology. Second, gut microbiota analysis was only performed in PD patients in this study. In this regard, as we did not have shotgun metagenomic data from control participants, which hampers us from performing functional analysis to compare the pathway differences which are highly correlated with AAAs. Further studies enrolling participants with concomitant information of fecal metagenomic shotgun sequencing data and plasma metabolite details are warranted to identify the specific bacterial species, which are highly correlated with AAAs, and the relevant functional pathways in the bacterial genomes. Third, although we showed that increased AAA metabolites were associated with gut microbiota, we cannot determine whether the correlations resulted from increased microbial production, enteric barrier dysfunction, or other mechanisms. Future research should include a concomitant leaky gut test. Fourth, we did not examine the inflammatory markers in feces or plasma to confirm our hypothesis that the AAA metabolites were associated with pro-inflammatory status in the gut and systemic circulation. Finally, we assessed cognitive function only with MMSE, a simple measurement of global cognitive function. Future studies should include detailed neuropsychological tests to evaluate individual cognitive domains, and participants with diverse cognitive dysfunction, to further assess the correlation between plasma AAA levels and cognitive decline in patients with PD.

In conclusion, our results revealed that AAA proteolytic metabolites in systemic circulation were upregulated in PD patients compared to controls. These metabolites were also associated with specific colonic bacterial species, and with motor symptom severity after adjusting for confounders, especially PAGln and Pcg. These findings suggest that dysregulation of microbial-associated proteolytic metabolism and AAA metabolites may contribute to PD pathophysiology.

## Methods

### Participants and clinical assessment

PD patients and neurologically normal controls were enrolled from the movement disorder clinic of National Taiwan University Hospital. PD was diagnosed according to the UK PD Society Brain Bank Clinical Diagnostic Criteria^33^. As controls, we enrolled neurologically unaffected persons accompanying the PD patients. Participants were excluded if they had renal or liver disease; history of inflammatory bowel disease, irritable bowel syndrome, colitis, or colon cancer; had used antibiotics or probiotic supplements within 3 months of enrollment; or were vegetarians. Constipation was diagnosed based on the standard Rome IV gastrointestinal disorders diagnostic criteria^34^. Among the enrolled participants, 96 patients with PD had undergone fecal shotgun metagenomic sequencing, which was previously published^35^.

The research protocol was reviewed by the Institutional Research Board Committee at National Taiwan University Hospital. All participants provided written informed consent before entering the study.

A comprehensive dietary history was collected using the Food Frequency Questionnaire (FFQ)^36^. Motor symptom severity was assessed during the “on” phase of PD using Hoehn and Yahr staging^37^ and Movement Disorder Society-Unified Parkinson’s Disease Rating Scale (MDS-UPDRS) part III motor scores^38^. Hoehn and Yahr stage of <3 was considered early-stage PD, and ≥3 as advanced-stage PD. Cognitive function was examined using the Mini-Mental State Examination (MMSE). Patients’ anti-PD medication dosage during the study period was converted into a levodopa equivalent daily dosage (LEDD).

### Measurement of plasma AAA using liquid chromatography-mass spectrometry

After participants fasted for at least 8 h, 10 µL venous blood was collected, centrifuged, and stored at −80 °C before further analysis. To quantify microbial metabolites, 500 ng/mL internal standards of all AAA-derived gut microbial metabolites, in 200 µL methanol, was added to 50 µL calibration standard solution and to 50 µL plasma, for protein precipitation. Samples were mixed with Geno/Grinder 2020 (OPS Diagnostics, LLC, NJ, USA) at 1000 rpm for 3 min, and then centrifuged at 15000 rcf for 5 min at 4 °C. We then filtered 200 µL of supernatant through a 0.2-µm filter (RC-4, Sartorius, Gottingen, Germany), followed by analysis with liquid chromatography-mass spectrometry (LC-MS).

LC separation was performed using an Agilent 1290 UHPLC system and Waters ACQUITY UPLC HSS T3 column (2.1 × 100 mm, 1.8 μm). The mobile phase was water with 2 mM ammonium acetate as solvent A, and water:acetonitrile (2:3, v-v) as solvent B. The flow rate was 0.3 mL/min, and the injection volume was 5 µL. The LC gradient was as follows: 0–1.5 min, 0–25% B; 1.5–4.5 min, 25% B; 4.5–4.6 min, 25–100% B; and 4.6–7 min, 100% B. Using an Agilent 6495 triple quadrupole mass spectrometer (Agilent Technologies, Santa Clara, CA) with an ESI source, we analysed the metabolites in negative ion mode, with a capillary voltage of 3500 V and nozzle voltage of 2000 V. For the dry gas and sheath gas, respectively, the temperatures were 290 °C and 325 °C, and the flow rates were 11 L/min and 10 L/min. The nebulizer was set to 40 psi. Spectra were acquired in multiple reaction monitoring mode. Analyte concentrations were calculated from calibration curves, with the peak area ratio of each analyte to its corresponding internal standard.

For data imputation, we imputed the missing values based on the average level of the individual AAA metabolite across all participants. If the mean metabolite measure across samples was >100,000, then zero was imputed, otherwise one half of the minimum measure for that sample was imputed^39^.

### Gut microbiota analysis using shotgun metagenomic sequencing

Gut microbiota analysis was only performed in patients with PD. Among 250 PD patients, the fecal shotgun metagenomic data derived from 96 PD patients was reported before with plasma samples collected at the same time^35^. The remaining 154 PD patients were newly recruited for assessing gut microbiota and plasma AAA metabolites in the current study. There was no significant batch effect as the beta diversity between these two batches of microbiota analyses did not reveal significant differences (Supplementary Fig. 4). Fecal sample collection, fecal DNA extraction, and gut microbiota analysis were performed^35^.

Fresh fecal samples were obtained from each participant and immediately preserved in stool specimen collection tubes containing DNA stabilizer (Sarstedt). These samples were promptly flash-frozen on dry ice and stored at −80 °C until further analysis. The total fecal DNA was extracted using the QIAamp DNA Stool Mini Kit (Qiagen, Hilden, Germany). To prepare the DNA for shotgun metagenomic sequencing, several steps were taken. First, the purity of the DNA was assessed using an Epoch Microplate Spectrophotometer (BioTek, USA). Then, the quantity of DNA was measured using the Quant-iT PicoGreen dsDNA Assay (ThermoFisher Scientific, USA). Subsequently, libraries were constructed using Illumina DNA Prep kits (Illumina, USA), targeting insert sizes of ~350 base pairs. The prepared libraries, with the specified insert sizes, were subjected to sequencing on an Illumina NovaSeq 6000 sequencer, utilizing S4 flow cells. For both case and control samples, the metagenomic Illumina paired-end reads underwent trimming, which was performed using Trimmomatic v0.39 with the following parameters: “PE -phred33 ILLUMINACLIP:TruSeq3-PE.fa:2:30:10 LEADING:12 TRAILING:12 SLIDINGWINDOW:4:15 MINLEN:36.”^40^.

The trimmed paired-end reads from the samples were subsequently subjected to a prokaryotic species profiling analysis using Kraken2 v2.0.6-beta 49^41^. To enhance the efficiency of this search, we employed the maxikraken2 database, which has a total size of 140 GB. This specialized database, constructed by Daniel Fischer at the Natural Resources Institute Finland and accessible at https://lomanlab.github.io/mockcommunity/mc_databases.html, was used in place of the default kraken2 database. Prior to investigating the relationship between gut microbiota and plasma AAA levels, we implemented a filtering process to exclude rare taxa, defined as those with a prevalence <10% among all samples and a mean abundance of <0.001%. Correlations between the relative abundance of gut microbiota and plasma AAA levels were assessed through Spearman’s rank sum tests, with Benjamini and Hochberg adjustment applied to account for multiple tests. Taxa with a *P-*value < 0.01 were selected for further analysis. To visualize the correlation between the relative abundance of gut microbiota and plasma levels of AAA, we utilized the R package “pheatmap”. All analyses were conducted using R software 3.4.1^42^.

The codes that were used in the R software were shown as below.

remove(list = ls())

library(tidyverse)

library(vegan)

library(pheatmap)

pd500 <- read.csv(“pd500_clean.csv”, header=TRUE, sep = “,”) ##read AAA data

kraken_pd500 <- read.csv(“kraken_pd500.csv”, header=TRUE, row.names=1, sep = “,”) %>% as.matrix() ##read abundance table

## filter out rare taxa (prevelance < 10%, mean abundance < 0.001%)

prev_idx <- sapply(1:1974,function(x) sum(kraken_pd500[x,]>0)) > 0.1*ncol(kraken_pd500)

abun_idx <- sapply(1:1974,function(x) mean(kraken_pd500[x,])) > 0.00001

kraken_pd500_f <- kraken_pd500[(prev_idx & abun_idx),]

## calculate spearman correlation

metabolite <- colnames(pd500)[3:11]

rho <- data.frame()

p_val <- data.frame()

for (i in 1:9) {

 for (j in 1: nrow(kraken_pd500_f)) {

  test <- cor.test(log(pd500[,i + 2]),log(kraken_pd500_f[j,]),method = “spearman”)

  rho[j,i] <- test$estimate

  p_val[j,i] <- test$p.value

 }

}

p_adj <- apply(p_val,2,function(x) p.adjust(x,“BH”))

colnames(rho) <- metabolite

colnames(p_val) <- paste0(metabolite,”_p”)

colnames(p_adj) <- paste0(metabolite,”_p_adj”)

result_simple <- rho

colnames(result_simple) <- metabolite

rownames(result_simple) <- rownames(kraken_pd500_f)

result <- cbind(rho,p_val,p_adj)

rownames(result) <- rownames(kraken_pd500_f)

##pheatmap version

##split data

result_pc <- result_simple[,6:9]

asterisk_pc <- cut(unlist(p_val[,6:9]), breaks=c(-Inf, 0.001, 0.01, 0.05, Inf),

     label=c(“***“, “**“, “*“, “”)) %>% matrix(., ncol=4)

result_phe <- result_simple[,1:5]

asterisk_phe <- cut(unlist(p_val[,1:5]), breaks=c(-Inf, 0.001, 0.01, 0.05, Inf),

     label=c(“***“, “**“, “*“, “”)) %>% matrix(., ncol=5)

##filter

idx_p_pc <- (p_val[,6] < 0.01) | (p_val[,7] < 0.01) | (p_val[,8] <0.01) | (p_val[,9] < 0.01)

result_pc <- result_pc[,2:4]

asterisk_pc <-asterisk_pc[,2:4]

pc_names <- lapply(

 rownames(result_pc),

 function(x) bquote(italic(.(x))))

pheatmap(result_pc[idx_p_pc,], color = colorRampPalette(c(“#33A1C9”, “white”, “brown3”))(50),

   fontsize_row=11, cluster_cols=FALSE, display_numbers = asterisk_pc[idx_p_pc,],

   labels_row = as.expression(pc_names))

idx_p_phe <- (p_val[,1] < 0.01) | (p_val[,2] < 0.01) | (p_val[,3] <0.01) | (p_val[,4] < 0.01) | (p_val[,5] < 0.01)

desired_order <- c(“PHE”,“PAA”, “PAGln”, “PPA”, “PLA”) ## Lin’s order 20230626

desired_res_phe <- result_phe[,desired_order]

pheatmap(result_phe[idx_p_phe,], color = colorRampPalette(c(“#33A1C9”, “white”, “brown3”))(50),

   fontsize_row=7, cluster_cols=FALSE, display_numbers = asterisk_phe[idx_p_phe,])

#labels_col could not change order

newnames <- lapply(

 rownames(desired_res_phe),

 function(x) bquote(italic(.(x))))

pheatmap(desired_res_phe[idx_p_phe,], color = colorRampPalette(c(“#33A1C9”, “white”, “brown3”))(50),

   fontsize_row=9, fontsize_col=11,

   cluster_cols=FALSE, display_numbers = asterisk_phe[idx_p_phe,],

   labels_row = as.expression(newnames)) # change spp names to italic

### Statistical analysis

Continuous variables are expressed as mean ± standard deviation, and categorical factors as numbers and percentages. We examined homogeneity of variances with Levene test. Variables were compared using Student’s *t*-test or analysis of variance if fulfilling Gaussian distribution, and using the nonparametric Mann-Whitney U test or Fisher’s exact test if the variables violated homoscedasticity or assumption of normality. The microbial AAA metabolite concentrations were log-transformed to achieve normal distribution for further analyses. A logistic regression model was applied to examine the relationship between AAA levels and PD occurrence, with adjustment for age and sex. We used the area under the receiver operating characteristic (ROC) curve (AUC) to quantify the model’s diagnostic performance for exploring the ability of AAA to distinguish between PD patients and controls. Spearman’s rank correlation was used to examine correlations between plasma AAA levels and disease severity. To examine associations between AAA and motor or cognitive symptom severity, we used multivariable linear regression models, with MDS-UPDRS part III and MMSE scores as dependent factors. The effect size of the model was estimated with Cohen’s f2. Independent variables included the log-transformed AAA levels, age, sex, disease duration, and LEDD. The correlations between the relative abundance of gut microbiota and plasma AAA levels were performed using Spearman’s rank sum tests with Benjamini and Hochberg adjustment for multiple tests. The statistical analyses were majorly performed in MedCalc software version 19.0.3 (MedCalc Software bvba, Ostend, Belgium) while the shotgun sequencing and microbiota related bioinformatic analysis were conducted using R software (3.4.1, R Foundation for Statistical Computing, Vienna, Austria). *P* < 0.05 was considered significant.

## Supplementary information

Supplementary tables and figures

Related Manuscript File

## Data Availability

The raw anonymized microbiome sequencing data for 117 participants who consented to open-access data sharing have been uploaded to the European Nucleotide Archive (https://www.ebi.ac.uk/ena/browser/home) under accession number PRJEB57770. Data for the remaining 37 participants are not publicly available due to consent restrictions but can be made available upon reasonable request to the corresponding author.

## Code availability

All code for data analysis associated with the current study is provided in the methods.
