# Supplementary material for: Plasma metabolites of aromatic amino acids associate with clinical severity and gut microbiota of Parkinson’s disease
Source: NPJ Parkinsons Dis. 2023 Dec 14;9:165. doi: 10.1038/s41531-023-00612-y (PMC10721883; doi:10.1038/s41531-023-00612-y)
Supplement: Supplementary file 1 — Supplementary tables and figures [file 41531_2023_612_MOESM1_ESM.pdf]

Supplementary Table 1. Dietary intakes of PD patients and controls in the current study.

Food and nutrients	Healthy controls (n=250) Median (IQR)	PD patients (n=250) Median (IQR)	*p* value
Meat, servings	0.83 (0.32, 1.81)	0.82 (0.38, 1.79)	0.89
Fish, servings	0.52 (0.31, 1.6)	0.51 (0.29, 1.5)	0.71
Eggs, servings	0.49 (0.23, 0.63)	0.44 (0.21, 0.70)	0.22
Soy, servings	1.32 (0.78, 0.68)	1.31 (0.65, 0.53)	0.81
Vegetables, servings	5.36 (2.13, 6.89)	5.43 (3.17, 7.03)	0.52
Fruits, servings	1.33 (0.82, 1.83)	1.29 (0.63, 2.03)	0.23
Nuts, servings	0.32 (0.11, 0.72)	0.29 (0.18, 0.65)	0.71
Energy, kcal	2454	2318	0.12
**Protein (% energy)**	13 (10, 14)	12 (10, 13)	0.10
**Fat (% energy)**	24 (19, 31)	24 (20, 32)	0.87
**Carbohydrate (% energy)**	64 (56, 67)	64 (57, 68)	0.58
**Dietary fiber, g**	28 (20, 34)	27 (21, 33)	0.51

Abbreviations: IQR = interquartile range.

Supplementary Table 2. Associations between plasma levels of AAA metabolites and the occurrence of PD.

Metabolites	Coefficient	OR	95% CI	*P* value
PHE	-0.78	0.46	0.06, 3.23	0.43
PAA	1.38	4.00	2.52, 6.53	<0.001***
PAGln	1.32	3.73	2.43, 5.84	<0.001***
PLA	1.11	3.03	1.25, 7.50	0.02*
PPA	0.56	1.75	1.36, 2.27	<0.001***
Pcs	0.83	2.29	1.74, 3.07	<0.001***
Pcg	0.73	2.07	1.65, 2.61	<0.001***
IS	1.11	3.02	1.79, 5.24	<0.001***

Multivariable logistic regression models were used to examine the associations between plasma metabolites and the risk of PD. Development of PD was set as dichotomous endpoint. The covariates included are age, sex, and log-transformed plasma metabolites.

CI, confidence interval; IS, indoxyl sulfate; OR, odds ratio; Pcg, p-Cresol glucuronide; Pcs, p-Cresol sulfate; PAA, phenylacetic acid; PAGln, phenylacetylglutamine; PHE, phenylalanine; PLA, phenyllactic acid; PPA, phenylpropionic acid.

*$P < 0.05$; **$P < 0.01$; ***$P < 0.001$.

Supplementary Table 3. Comparison of plasma individual AAA level between PD patients using different types of anti-parkinsonian medications.

		PHE (ug/ml)	PAA (ug/ml)	PAGln (ug/ml)	PLA (ug/ml)	PPA (ug/ml)	Pcs (ug/ml)	Pcg (ug/ml)	IS (ug/ml)
Dopaminergic medication	Non-user	9744 ± 2107	137 ± 80	1211 ± 604	31 ± 21	65 ± 114	4008 ± 2722	56 ± 70	1236 ± 790
	User	9628 ± 2352	98 ± 81	898 ± 1218	30 ± 17	59 ± 129	2761 ± 2916	32 ± 53	1062 ± 1665
	P value	0.35	<0.001***	<0.001***	0.36	0.90	<0.001***	<0.001***	<0.001***
COMT inhibitor	Non-user	9563 ± 2227	90 ± 78	764 ± 749	29 ± 17	58 ± 136	2483 ± 2418	29 ± 50	886 ± 609
	User	9789 ± 2283	138 ± 81	1289 ± 1191	32 ± 20	65 ± 108	4060 ± 3132	54 ± 70	1382 ± 1832
	P value	0.36	<0.001***	<0.001***	0.12	0.34	<0.001***	<0.001***	<0.001***
MAO-B inhibitor	Non-user	9817 ± 2445	108 ± 82	1021 ± 1264	31 ± 16	70 ± 139	3019 ± 3148	41 ± 70	1129 ± 1763
	User	9516 ± 2015	120 ± 83	1028 ± 670	30 ± 21	52 ± 102	3540 ± 2579	41 ± 51	1135 ± 760
	P value	0.64	0.13	0.08	0.49	0.28	0.02*	0.13	0.08
Amantadine	Non-user	9603 ± 2218	78 ± 68	742 ± 1367	29 ± 17	46 ± 87	2068 ± 3039	27 ± 67	928 ± 1977
	User	9721 ± 2281	137 ± 84	1206 ± 671	31 ± 21	71 ± 140	4037 ± 2530	51 ± 56	1264 ± 766

	P value	0.52	<0.001***	<0.001***	0.26	0.01*	<0.001***	<0.001***	<0.001***
Trihexyphenidyl	Non-user	9728 ± 2258	114 ± 76	1044 ± 1015	30 ± 19	64 ± 130	3367 ± 2882	43 ± 63	1176 ± 1463
	User	9377 ± 2232	111 ± 114	913 ± 1093	32 ± 16	45 ± 73	2699 ± 2971	30 ± 49	889 ± 738
	P value	0.21	0.09	0.01**	0.42	0.41	0.04*	0.03*	0.01**

CI, confidence interval; COMT inhibitor, catechol-O-methyltransferase inhibitor; IS, indoxyl sulfate; MAO-B inhibitor, monoamine oxidase-B inhibitor; Pcg, p-Cresol glucuronide; Pcs, p-Cresol sulfate; PAA, phenylacetic acid; PAGln, phenylacetylglutamine; PHE, phenylalanine; PLA, phenyllactic acid; PPA, phenylpropionic acid.

*$P < 0.05$; **$P < 0.01$; ***$P < 0.001$.

Supplementary table 4. Multiple linear regression analyses of individual AAA metabolite and clinical disease severity with adjustment for potential confounding covariates in patients with PD.

Metabolite	Covariates	MDS-UPDRS part III scores			MMSE scores		
		Coefficient	95% CI	*P* value	Coefficient	95% CI	*P* value
PHE	Age	-0.10	-0.26, 0.05	0.199	0.03	-0.01, 0.07	0.174
	Sex	0.04	-2.51, 2.58	0.977	-0.33	-1.01, 0.36	0.348
	Constipation	-0.55	-3.02, 1.92	0.659	0.39	-0.27, 1.05	0.243
	Dopaminergic supplement	0.31	-3.10, 3.73	0.856	-0.48	-1.40, 0.43	0.300
	Monoamine oxidase-B inhibitor	-2.84	-5.50, -0.18	0.036*	0.46	-0.26, 1.17	0.209
	Catechol-O-methyltransferase	3.69	0.40, 6.98	0.028*	-1.32	-2.20, -0.44	0.003**
	Trihexyphenidyl	-0.68	-4.43, 3.08	0.723	0.40	-0.60, 1.41	0.430
	Amantadine	3.80	1.04, 6.57	0.007**	-0.51	-1.25, 0.23	0.175
PAGln	Age	-0.10	-0.26, 0.06	0.207	0.03	-0.02, 0.07	0.209
	Sex	0.08	-2.45, 2.61	0.949	-0.33	-1.02, 0.35	0.338
	Constipation	-0.58	-3.03, 1.86	0.640	0.34	-0.33, 1.00	0.318

	Dopaminergic supplement	0.48	-2.91, 3.88	0.780	-0.49	-1.41, 0.42	0.291
	Monoamine oxidase-B inhibitor	-2.95	-5.59, -0.31	0.029*	0.48	-0.23, 1.20	0.183
	Catechol-O-methyltransferase	2.61	-0.82, 6.03	0.135	-1.30	-2.22, -0.37	0.006**
	Trihexyphenidyl	-0.58	-4.31, 3.15	0.760	0.43	-0.58, 1.44	0.404
	Amantadine	2.39	-0.68, 5.45	0.126	-0.48	-1.31, 0.35	0.256
PLA	Age	-0.09	-0.25, 0.06	0.239	0.03	-0.02, 0.07	0.219
	Sex	-0.01	-2.57, 2.55	0.994	-0.35	-1.03, 0.34	0.320
	Constipation	-0.41	-2.89, 2.07	0.747	0.30	-0.36, 0.97	0.372
	Dopaminergic supplement	0.37	-3.06, 3.79	0.832	-0.48	-1.40, 0.43	0.300
	Monoamine oxidase-B inhibitor	-2.93	-5.60, -0.27	0.031*	0.49	-0.22, 1.20	0.178
	Catechol-O-methyltransferase	3.69	0.39, 6.99	0.029*	-1.34	-2.22, -0.46	0.003**
	Trihexyphenidyl	-0.92	-4.69, 2.85	0.631	0.41	-0.60, 1.43	0.420
	Amantadine	3.80	1.01, 6.58	0.008**	-0.53	-1.28, 0.21	0.159
PAA	Age	-0.09	-0.25, 0.06	0.245	0.03	-0.02, 0.07	0.215
	Sex	0.05	-2.50, 2.61	0.968	-0.34	-1.02, 0.35	0.334

	Constipation	-0.32	-2.77, 2.14	0.801	0.32	-0.34, 0.98	0.340
	Dopaminergic supplement	0.37	-3.06, 3.79	0.833	-0.48	-1.40, 0.43	0.299
	Monoamine oxidase-B inhibitor	-2.97	-5.63, -0.30	0.029*	0.48	-0.23, 1.20	0.184
	Catechol-O-methyltransferase	3.52	0.12, 6.92	0.043*	-1.36	-2.27, -0.45	0.004**
	Trihexyphenidyl	-0.80	-4.56, 2.96	0.675	0.44	-0.57, 1.44	0.395
	Amantadine	3.57	0.59, 6.55	0.019*	-0.56	-1.36, 0.23	0.164
PPA	Age	-0.09	-0.25, 0.07	0.267	0.03	-0.02, 0.07	0.214
	Sex	0.07	-2.48, 2.63	0.954	-0.33	-1.02, 0.35	0.338
	Constipation	-0.25	-2.70, 2.21	0.842	0.33	-0.33, 0.98	0.329
	Dopaminergic supplement	0.30	-3.13, 3.73	0.863	-0.49	-1.41, 0.43	0.295
	Monoamine oxidase-B inhibitor	-2.90	-5.59, -0.21	0.035*	0.49	-0.23, 1.21	0.185
	Catechol-O-methyltransferase	3.70	0.39, 7.00	0.029*	-1.33	-2.22, -0.45	0.003**
	Trihexyphenidyl	-0.77	-4.54, 3.00	0.689	0.44	-0.57, 1.45	0.395
	Amantadine	3.76	0.94, 6.59	0.009**	-0.53	-1.28, 0.23	0.172
Pcs	Age	-0.09	-0.25, 0.06	0.240	0.03	-0.02, 0.07	0.214

	Sex	0.33	-2.23, 2.88	0.800	-0.31	-1.00, 0.37	0.370
	Constipation	-0.61	-3.08, 1.86	0.626	0.30	-0.36, 0.96	0.374
	Dopaminergic supplement	0.37	-3.04, 3.77	0.832	-0.49	-1.40, 0.43	0.300
	Monoamine oxidase-B inhibitor	-2.99	-5.64, -0.34	0.027*	0.48	-0.23, 1.19	0.185
	Catechol-O-methyltransferase	3.22	-0.11, 6.55	0.058	-1.37	-2.27, -0.47	0.003**
	Trihexyphenidyl	-0.64	-4.38, 3.11	0.738	0.45	-0.56, 1.46	0.383
	Amantadine	3.02	0.10, 5.94	0.043*	-0.59	-1.37, 0.20	0.143
Pcg	Age	-0.10	-0.26, 0.05	0.199	0.03	-0.02, 0.07	0.221
	Sex	0.27	-2.27, 2.81	0.835	-0.32	-1.01, 0.36	0.352
	Constipation	-0.54	-2.98, 1.91	0.667	0.31	-0.35, 0.97	0.349
	Dopaminergic supplement	0.60	-2.81, 4.01	0.728	-0.48	-1.40, 0.44	0.308
	Monoamine oxidase-B inhibitor	-2.89	-5.54, -0.25	0.032*	0.49	-0.23, 1.20	0.180
	Catechol-O-methyltransferase	3.24	-0.06, 6.55	0.055	-1.35	-2.25, -0.46	0.003**
	Trihexyphenidyl	-0.59	-4.32, 3.15	0.757	0.44	-0.56, 1.45	0.386
	Amantadine	2.98	0.10, 5.86	0.043*	-0.56	-1.34, 0.22	0.156

IS	Age	-0.10	-0.26, 0.05	0.201	0.03	-0.01, 0.07	0.189
	Sex	-0.05	-2.60, 2.49	0.967	-0.31	-1.00, 0.37	0.366
	Constipation	-0.55	-3.01, 1.91	0.661	0.37	-0.29, 1.03	0.275
	Dopaminergic supplement	0.20	-3.21, 3.61	0.907	-0.47	-1.38, 0.45	0.316
	Monoamine oxidase-B inhibitor	-2.93	-5.58, -0.28	0.030*	0.48	-0.23, 1.19	0.186
	Catechol-O-methyltransferase	3.06	-0.31, 6.44	0.075	-1.23	-2.14, -0.33	0.008**
	Trihexyphenidyl	-0.55	-4.30, 3.21	0.775	0.40	-0.61, 1.40	0.440
	Amantadine	3.10	0.20, 6.01	0.036*	-0.41	-1.19, 0.37	0.299

CI, confidence interval; IS, indoxyl sulfate; MDS-UPDRS, Movement Disorder Society Unified PD Rating Scale; MMSE, Mini-Mental State Examination; Pcg, *p*-Cresol glucuronide; Pcs, *p*-Cresol sulfate; PAA, phenylacetic acid; PAGln, phenylacetylglutamine; PHE, phenylalanine; PLA, phenyllactic acid; PPA, phenylpropionic acid. *$P < 0.05$; **$P < 0.01$.

Supplementary Table 5. Associations between plasma levels of AAA metabolites and the occurrence of constipation symptom in PD patients.

	Coefficient	95% CI	*P* value
PHE	0.16	0.03, 0.29	0.02*
PAGln	0.11	0.01, 0.21	0.03*
PLA	0.07	0.01, 0.12	0.02*
PAA	0.07	-0.04, 0.18	0.20
PPA	-0.05	-0.17, 0.08	0.47
Pcs	0.15	0.04, 0.26	0.008**
Pcg	0.11	-0.01, 0.22	0.06
IS	0.13	0.01, 0.24	0.03*

Analysis of covariance was performed to analyze the associations between constipation and plasma AAA metabolites adjusting for age, sex, creatinine level, disease severity and usage of antiparkinsonian medication.

CI, confidence interval; IS, indoxyl sulfate; Pcg, p-Cresol glucuronide; Pcs, p-Cresol sulfate; PAA, phenylacetic acid; PAGln, phenylacetylglutamine; PHE, phenylalanine; PLA, phenyllactic acid; PPA, phenylpropionic acid.

*$P < 0.05$; **$P < 0.01$.

Supplementary Figure 1

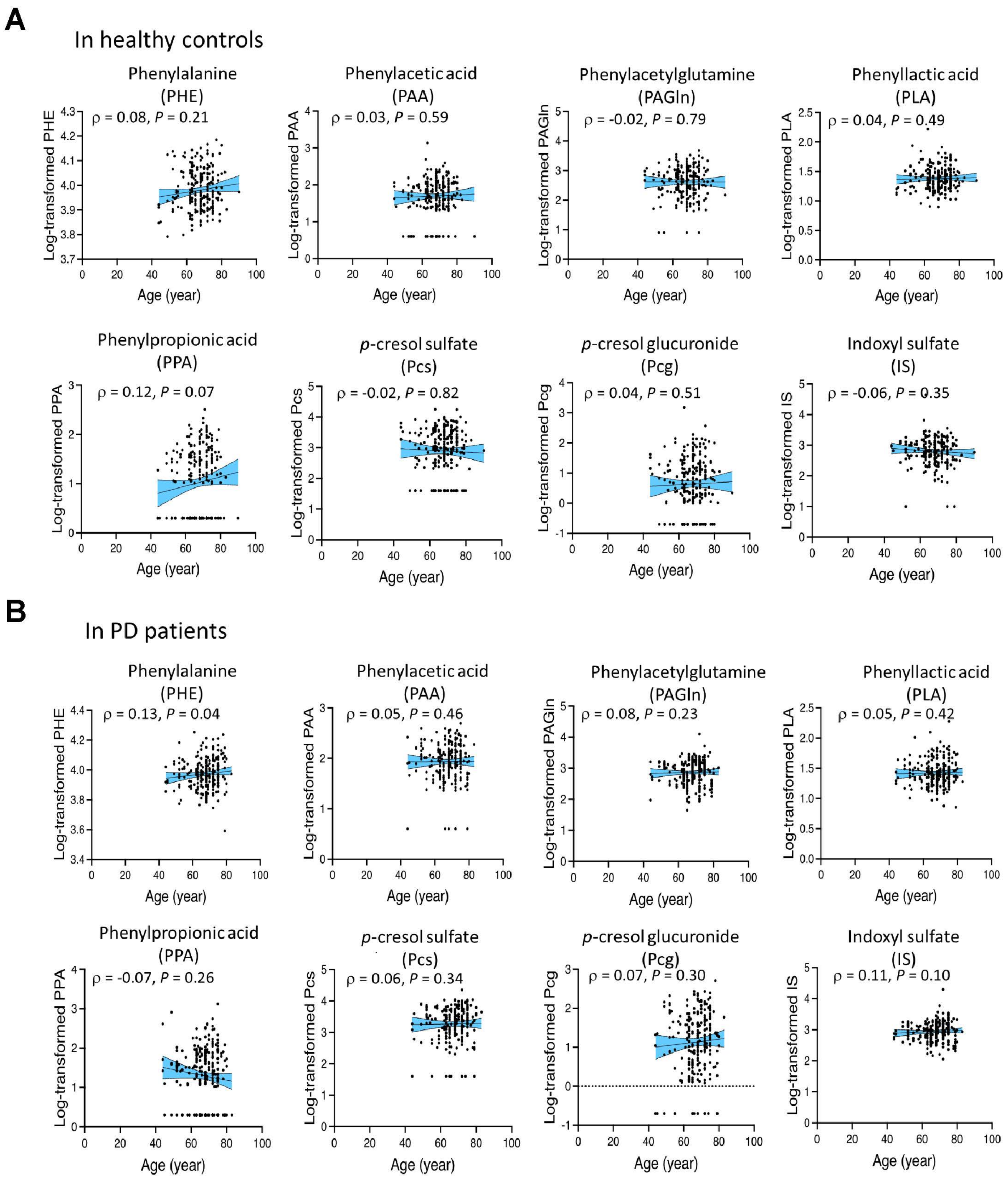

Supplementary Figure 1. Scatter plots illustrate the associations between age and plasma metabolites among controls (A) and patients with PD (B).

Supplementary Figure 2

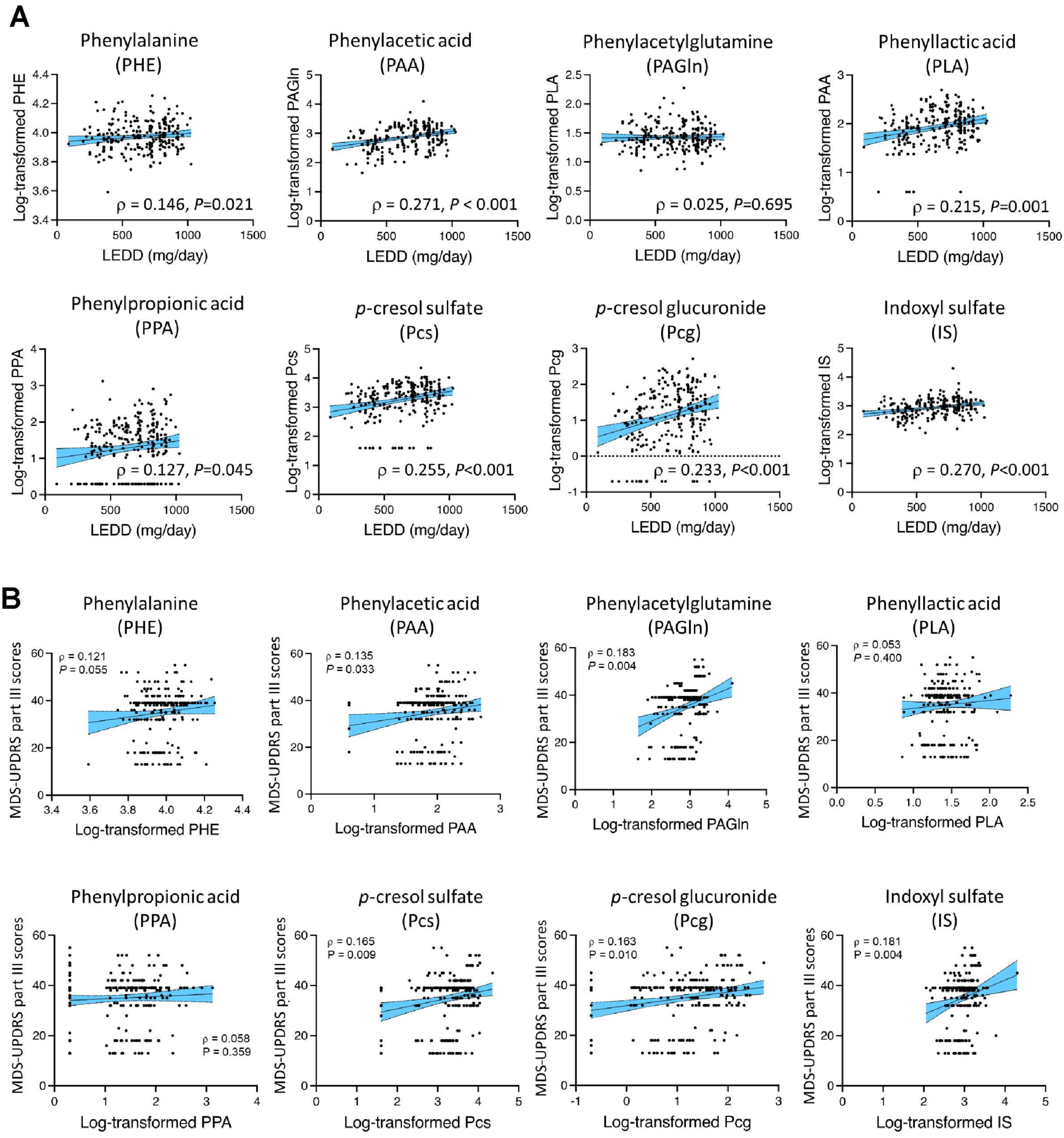

Supplementary Figure 2. Scatter plots show correlations between microbial metabolites and (A) levodopa equivalent daily dose (LEDD), and (B) motor symptom severity measured as MDS-UPDRS part III scores in patients with PD.

Supplementary Figure 3

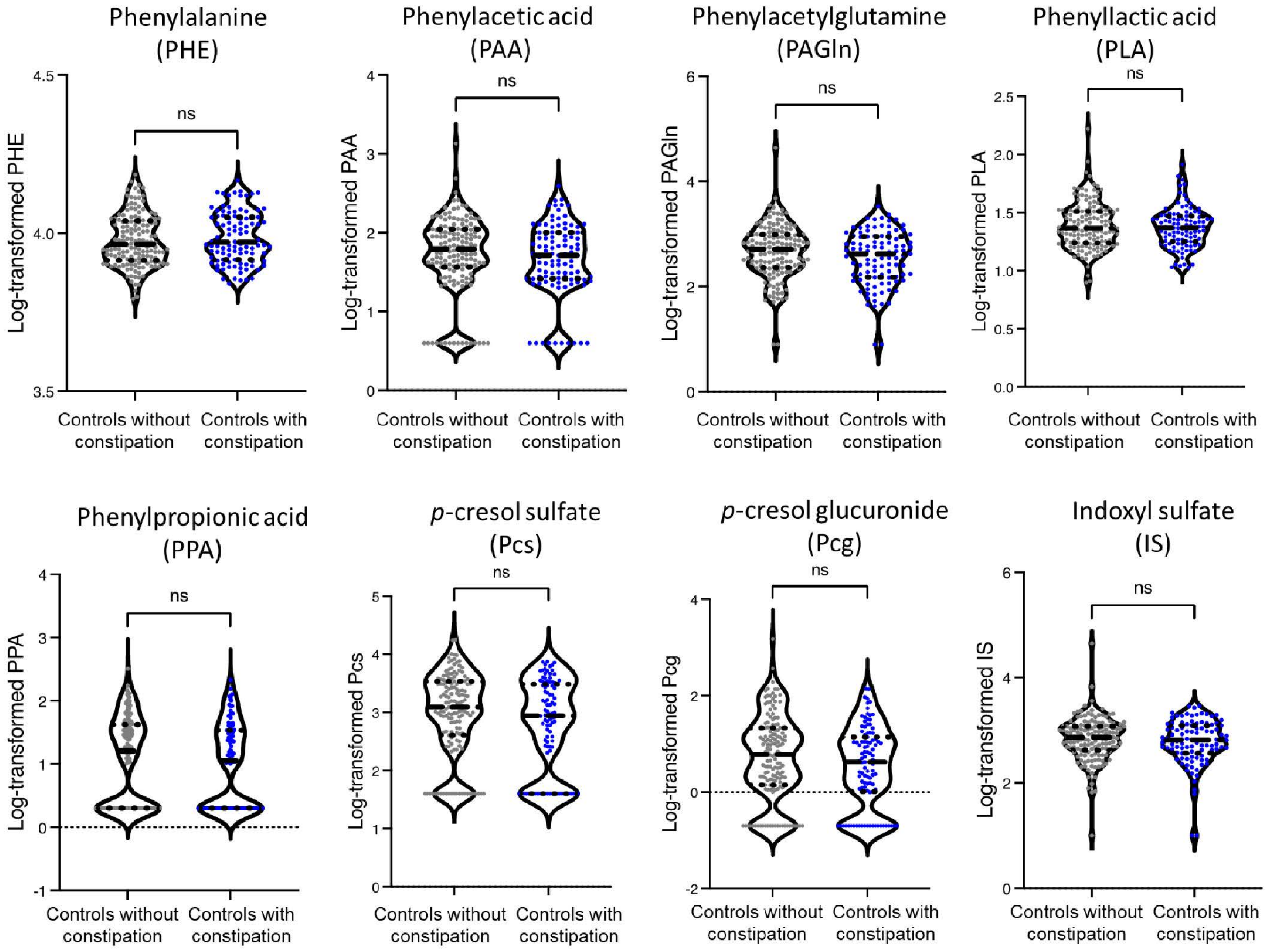

Supplementary Figure 3. Comparison of microbial metabolites between constipated and non-constipated normal controls. Violin plot shows the plasma levels of microbial metabolites in healthy controls. Solid and dashed horizontal lines indicate the median value and interquartile range, respectively. ns, non-specific.

Supplementary Figure 4

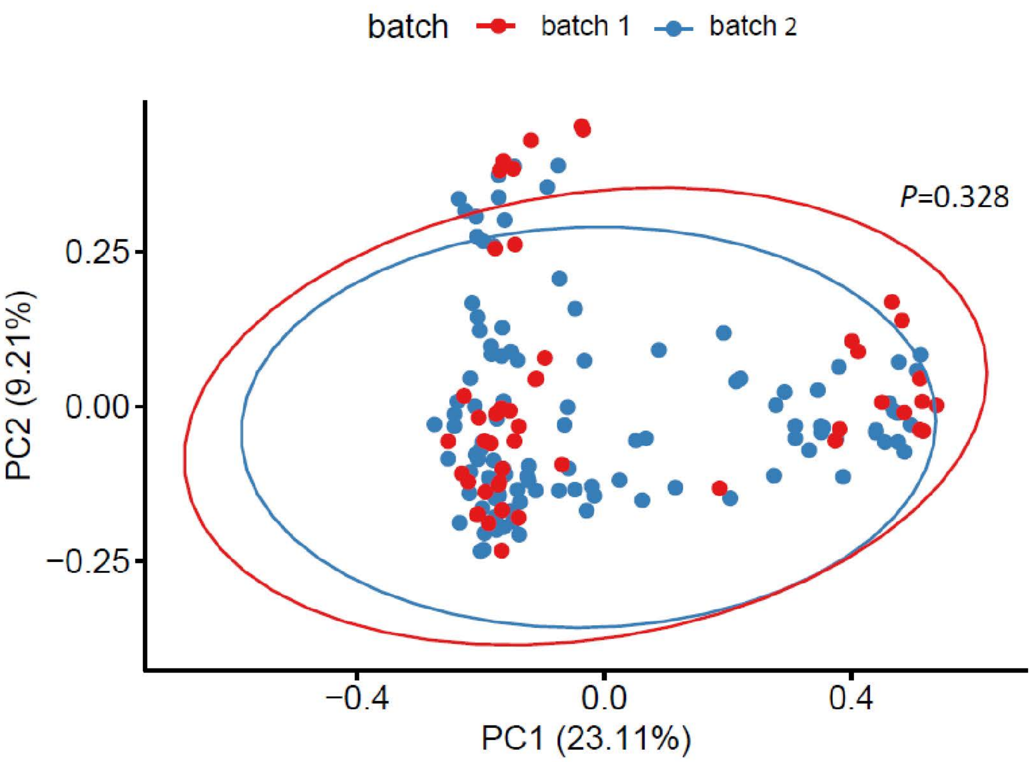

Supplementary Figure 4. The PCoA plot of bacterial β-diversity indices of the fecal microbiome in the PD patients from batch 1 and batch 2.
