# Supplementary material for: Plasma metabolites of aromatic amino acids associate with clinical severity and gut microbiota of Parkinson’s disease
Source: NPJ Parkinsons Dis. 2023 Dec 14;9:165. doi: 10.1038/s41531-023-00612-y (PMC10721883; doi:10.1038/s41531-023-00612-y)
Supplement: Supplementary file 2 — Related Manuscript File [file 41531_2023_612_MOESM2_ESM.pdf]

nature portfolio

Corresponding author(s): CH Kuo and CH Lin

Last updated by author(s): Nov 16, 2023

Reporting Summary

Nature Portfolio wishes to improve the reproducibility of the work that we publish. This form provides structure for consistency and transparency in reporting. For further information on Nature Portfolio policies, see our Editorial Policies and the Editorial Policy Checklist.

Statistics

For all statistical analyses, confirm that the following items are present in the figure legend, table legend, main text, or Methods section.

n/a	Confirmed	
☐	☒	The exact sample size (*n*) for each experimental group/condition, given as a discrete number and unit of measurement
☐	☒	A statement on whether measurements were taken from distinct samples or whether the same sample was measured repeatedly
☐	☒	The statistical test(s) used AND whether they are one- or two-sided<br>*Only common tests should be described solely by name; describe more complex techniques in the Methods section.*
☐	☒	A description of all covariates tested
☐	☒	A description of any assumptions or corrections, such as tests of normality and adjustment for multiple comparisons
☐	☒	A full description of the statistical parameters including central tendency (e.g. means) or other basic estimates (e.g. regression coefficient) AND variation (e.g. standard deviation) or associated estimates of uncertainty (e.g. confidence intervals)
☐	☒	For null hypothesis testing, the test statistic (e.g. F, t, r) with confidence intervals, effect sizes, degrees of freedom and P value noted<br>*Give P values as exact values whenever suitable.*
☐	☒	For Bayesian analysis, information on the choice of priors and Markov chain Monte Carlo settings
☐	☒	For hierarchical and complex designs, identification of the appropriate level for tests and full reporting of outcomes
☐	☒	Estimates of effect sizes (e.g. Cohen's *d*, Pearson's *r*), indicating how they were calculated

Our web collection on statistics for biologists contains articles on many of the points above.

Software and code

Policy information about availability of computer code

Data collection	The raw anonymized microbiome sequencing data were uploaded to the European Nucleotide Archive database (https://www.ebi.ac.uk/ena/browser/home) with the accession number PRJEB57770.
Data analysis	The statistical analyses were majorly performed in MedCalc software version 19.0.3 (MedCalc Software bvba, Ostend, Belgium) while the shotgun sequencing and microbiota related bioinformatic analysis were conducted using R software (3.4.1, R Foundation for Statistical Computing, Vienna, Austria).

For manuscripts utilizing custom algorithms or software that are central to the research but not yet described in published literature, software must be made available to editors and reviewers. We strongly encourage code deposition in a community repository (e.g. GitHub). See the Nature Portfolio guidelines for submitting code & software for further information.

Data

Policy information about availability of data

All manuscripts must include a data availability statement. This statement should provide the following information, where applicable:

- Accession codes, unique identifiers, or web links for publicly available datasets
- A description of any restrictions on data availability
- For clinical datasets or third party data, please ensure that the statement adheres to our policy

Provide your data availability statement here.

Research involving human participants, their data, or biological material

Policy information about studies with human participants or human data. See also policy information about sex, gender (identity/presentation), and sexual orientation and race, ethnicity and racism.

Reporting on sex and gender	The age and sex were comparable between patients with Parkinson's disease and control participants in the current study.
Reporting on race, ethnicity, or other socially relevant groupings	All participants were Taiwanese.
Population characteristics	This study enrolled a total of 500 participants, including 250 PD patients (67.4±7.7 years old, 65.6% male) and 250 normal controls (67.4±7.5 years old, 66.0% male). The participants' clinical characteristics are listed in table 1.
Recruitment	PD patients and neurologically normal controls were enrolled from the movement disorder clinic of National Taiwan University Hospital.
Ethics oversight	The research protocol was reviewed by the Institutional Research Board Committee at National Taiwan University Hospital.

Note that full information on the approval of the study protocol must also be provided in the manuscript.

Field-specific reporting

Please select the one below that is the best fit for your research. If you are not sure, read the appropriate sections before making your selection.

☒ Life sciences ☐ Behavioural & social sciences ☐ Ecological, evolutionary & environmental sciences

For a reference copy of the document with all sections, see nature.com/documents/nr-reporting-summary-flat.pdf

Life sciences study design

All studies must disclose on these points even when the disclosure is negative.

Sample size	The current sample size with a total number of 500 participants was the largest sample size in the field of gut metabolite assay of Parkinson's disease.
Data exclusions	No data were excluded from the analysis.
Replication	Measurement of plasma gut metabolites using liquid chromatography-mass spectrometry were replicated successfully in house.
Randomization	This is not a clinical trial study and, therefore, there is no randomization.
Blinding	This is not a clinical trial study and, therefore, there is no blinding.

Reporting for specific materials, systems and methods

We require information from authors about some types of materials, experimental systems and methods used in many studies. Here, indicate whether each material, system or method listed is relevant to your study. If you are not sure if a list item applies to your research, read the appropriate section before selecting a response.

Materials & experimental systems

n/a	Involved in the study	
☒	☐	Antibodies
☒	☐	Eukaryotic cell lines
☒	☐	Palaeontology and archaeology
☒	☐	Animals and other organisms
☐	☒	Clinical data
☒	☐	Dual use research of concern
☒	☐	Plants

Methods

n/a	Involved in the study	
☒	☐	ChIP-seq
☒	☐	Flow cytometry
☒	☐	MRI-based neuroimaging

Clinical data

Policy information about clinical studies

All manuscripts should comply with the ICMJE guidelines for publication of clinical research and a completed CONSORT checklist must be included with all submissions.

Clinical trial registration	N/A, the study is not a clinical trial.
Study protocol	Plasma AAA were measured using liquid chromatography-mass spectrometry
Data collection	A comprehensive dietary history was collected using the Food Frequency Questionnaire (FFQ). Motor symptom severity was assessed during the "on" phase of PD using Hoehn and Yahr staging and Movement Disorder Society-Unified Parkinson's Disease Rating Scale (MDS-UPDRS) part III motor scores. Hoehn and Yahr stage of <3 was considered early-stage PD, and ≥3 as advanced-stage PD. Cognitive function was examined using the Mini-Mental State Examination (MMSE). Patients' anti-PD medication dosage during the study period was converted into a levodopa equivalent daily dosage (LEDD).
Outcomes	Plasma AAA levels

Plants

Seed stocks	N/A
Novel plant genotypes	N/A
Authentication	N/A
